# Brothers in Arms: Structure, Assembly and Function of *Arenaviridae* Nucleoprotein

**DOI:** 10.3390/v12070772

**Published:** 2020-07-17

**Authors:** Nicolas Papageorgiou, Maria Spiliopoulou, Thi-Hong Van Nguyen, Afroditi Vaitsopoulou, Elsie Yekwa Laban, Karine Alvarez, Irene Margiolaki, Bruno Canard, François Ferron

**Affiliations:** 1 Architecture et Fonction des Macromolécules Biologiques, CNRS - UMR 7257, Polytech Case 925, 13009 Marseille, France; Nicolas.Papageorgiou@afmb.univ-mrs.fr (N.P.); Thi-Hong-Van.Nguyen@afmb.univ-mrs.fr (T.-H.V.N.); elsielaban@gmail.com (E.Y.L.); Karine.Alvarez@afmb.univ-mrs.fr (K.A.); bruno.canard@afmb.univ-mrs.fr (B.C.); 2 Aix-Marseille Université, AFMB UMR 7257, 13288 Marseille, France; afi.5@hotmail.com; 3 Section of Genetics, Cell Biology and Development, Department of Biology, University of Patras, GR-26500 Patras, Greece Patras, Greece; mary.spiliopoulou94@gmail.com (M.S.); irenemargiolaki1@gmail.com (I.M.); 4 School of Life & Health Sciences, Aston University, Aston Triangle, Birmingham B4 7ET, UK; 5 New Mexico State University, P.O Box 30001, Las Cruces, NM 88003-8001, USA; 6 European Virus Bioinformatics Center, Leutragraben 1, 07743 Jena, Germany

**Keywords:** *Arenaviridae*, *Bunyavirales*, emerging diseases, exonuclease, nucleoprotein, structure

## Abstract

*Arenaviridae* is a family of viruses harbouring important emerging pathogens belonging to the *Bunyavirales* order. Like in other segmented negative strand RNA viruses, the nucleoprotein (NP) is a major actor of the viral life cycle being both (i) the necessary co-factor of the polymerase present in the L protein, and (ii) the last line of defence of the viral genome (vRNA) by physically hiding its presence in the cytoplasm. The NP is also one of the major players interfering with the immune system. Several structural studies of NP have shown that it features two domains: a globular RNA binding domain (NP-core) in its N-terminal and an exonuclease domain (ExoN) in its C-terminal. Further studies have observed that significant conformational changes are necessary for RNA encapsidation. In this review we revisited the most recent structural and functional data available on *Arenaviridae* NP, compared to other *Bunyavirales* nucleoproteins and explored the structural and functional implications. We review the variety of structural motif extensions involved in NP–NP binding mode. We also evaluate the major functional implications of NP interactome and the role of ExoN, thus making the NP a target of choice for future vaccine and antiviral therapy.

## 1. Introduction

Arenaviruses are zoonotic viruses that cause chronic infections in rodents, which constitute a reservoir of human pathogens across the world. The *Arenaviridae* family was recently reclassified into the *Bunyavirales*, a viral order that includes several major human pathogens, including the Rift Valley Fever virus (RVFV), Hantaan virus (HTNV) and Crimean Congo Haemorrhagic Fever virus (CCHFV). *Arenaviridae* regroups *Mammarenavirus*, *Reptarenavirus*, *Hartmanivirus*, and *Antennavirus* [1,2]. Mammarenaviruses are further classified into two groups based on geography and phylogeny: the Old World (OW) arenaviruses and the New World (NW) arenaviruses, itself divided into clades A, B, C and D. Several of the *Mammarenavirus* are responsible for viral haemorrhagic fevers (VHFs) in humans: OW Lassa virus (LASV) and NW clade B: Junin virus (JUNV), Machupo virus (MACV), Guanarito virus (GTOV), Sabia virus (SABV), and Chapare virus (CHAV). The OW prototype Lymphocytic choriomeningitis virus (LCMV) can cause nervous disorders like meningitis and hearing loss [3,4], and is responsible for a large number of miscarriages [5,6] due to neonatal infections and health complications for immune-compromised individuals [7,8,9]. Meanwhile, NW Pichinde virus (PICV) from the prototype Tacaribe Virus (TCRV) complex is non-pathogenic for humans and animals. Recently, OW arenaviruses have been isolated in Asia from mice, shrews and black rats, expanding host variety and geographic distribution of *Mammarenavirus* [10,11,12,13], and the list of human pathogens [14]. In recent years, repeated LASV outbreaks pointed out the major public health concerns in their regions of endemicity and surroundings [15,16,17,18,19,20] not only due to the severe acute disease and high mortality rates, but also to the long-term sequelae responsible for significant social and economic burdens [21,22]. Finally, the frequency of imported cases in Europe and the USA have increased in the last few years, illustrating the possibility of imported cases of haemorrhagic fever of both OW and NW arenaviruses [23,24,25,26].

Arenaviruses are enveloped viruses containing a segmented negative-sense single-stranded RNA genome (sNSV). Apart from the tri-segmented antennaviruses genus, the RNA genome (vRNA) is comprised of two segments: a large segment (L) of around 7.2 kb and a small segment (S) of around 3.4 kb. Each segment uses an ambisense coding strategy to direct synthesis of two proteins in opposite orientation separated by an intergenic region (IGR). The L segment encodes the large protein L (~ 200 kDa) and a small protein Z (~ 11 kDa) that functions as the matrix protein of the virion. The S segment encodes the multi-functional nucleoprotein (see below) NP (~ 63 kDa) and the glycoprotein precursor (GPC; 75 kDa), that will give after post-translational cleavage, GP1 (40 to 46 kDa), GP2 (35 kDa) and SSP a transmembrane stable signal peptide. The mature glycoprotein (GP) complex on the viral surface is a trimer of heterotrimers composed of GP1/GP2 and SSP. The IGR is thought to fold into secondary structures, which lead to viral messenger RNA (mRNA) transcription termination [27]. The RNA genome (and complementary) is always encapsulated in a polymer of NP forming the ribonucleoprotein complex (RNP). The 5’ and 3’ ends of each segment contain conserved untranslated regions (UTR) that are complementary to each other, forming a panhandle structure at the end of the viral genome [28,29] on which binds the L. The whole (RNP-L) constitutes the replication–transcription complexes (RTC) and NP is a necessary co-factor of L [30]. Like all other *Bunyavirales* nucleoproteins, NPs are the most abundant viral proteins both in infected cells and virions. They are *de facto* the main structural and multi-functional component of the viral cycle but, unlike the other nucleoproteins, they are also multi-domain proteins. By coating the vRNA (or anti-genomic), they passively protect the viral genome from degradation, avoid formation of dsRNA between viral RNAs of opposite polarity, and compact the RNA into RNPs. Moreover, they are responsible for generating a significant interference in the transduction pathway signalling cellular infection by recruiting several host proteins. Finally, through the unique presence of an active exonuclease (ExoN), it actively degradates dsRNA, contributing to the silencing of innate immunity and to a yet unclear process seemingly playing a role in replication.

In infected cells, NP binds to vRNA but neither to viral mRNAs nor to cellular mRNA; however, when recombinant NPs are expressed in bacteria, they are observed to bind to host RNAs forming structures reminiscent of the viral RNP structure. This RNP formation suggests that the polymerisation process is tightly regulated in the infected cell. 

In this review, we revisit the last structural and functional data available on *Arenaviridae* NP as well as other closely related nucleoproteins and explore their structural/functional implications.

## 2. Arenavirus Nucleoprotein Architecture and Structure from Atomic Structures to Observation 

### 2.1. Architecture and Full-Length Structure of NP

**Figure 1:**
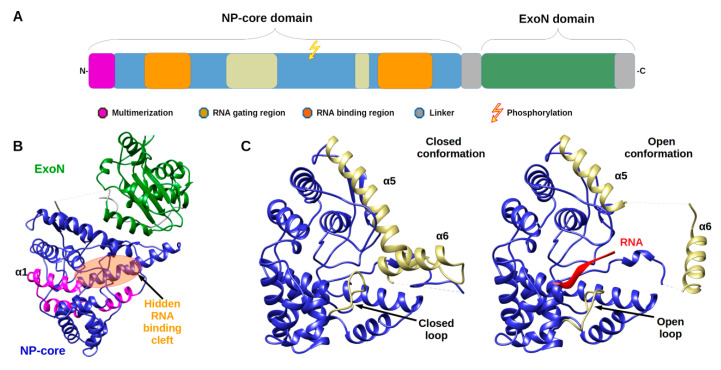
Architecture of *Arenaviridae* nucleoprotein (NP) and Nucleoprotein structures. (**A**) Annotated schematic of NP architecture. Colour code of annotation is in caption. (**B**) Structure of full-length NP (PDB 3MWP) represented in ribbon. NP-core domain is in blue and the exonuclease domain (ExoN) in green, the proposed multimerization arm of the NP-core in pink, the hidden RNA binding cleft highlighted in orange circle. (**C**) Structure of NP-core domain (blue/ kaki) in open and closed conformation focus on the RNA binding cleft. Left panel shows the RNA binding cleft of the 3MWP structure. Right panel presents the corresponding domain of the structure 3T5Q with RNA (red ribbon). Comparison of these two structures shows that in the absence of RNA, the cavity is closed by the α5 and α6 helix shown in kaki as well as by the loop (residues 234–245) shown also in kaki. The α5 and α6 helix as well as the loop are displaced in the case of the 3T5Q structure, permitting the adsorption of the viral RNA. All structural figures and movies were done using UCSF chimera [31].

Arenavirus NPs are involved in several critical functions for the virus life cycle: transcription/replication, genome/anti genome protection (both passive and active) and genome packaging. These functions are reflected in the structural architecture of NP as a two domain protein, an amino terminal domain (N-terminal) involved in polymerisation and viral RNA protection and a carboxy terminal domain (C-terminal) involved in degrading dsRNA, a marker of viral infection. The latter domain is surrounded by two flexible linkers that impact high resolution structural studies (Figure 1). 

The monomeric full-length NP crystal structure of Lassa virus [32,33] presents a two domain protein separated by a flexible linker (of 30 amino acids) uncharacterised in structure due to its intrinsic flexibility (Figure 1b). Both domains are in tight contact with each other, and their respective position is likely due to the crystal packing.

The N-terminal domain is a globular α/β domain composed of 14 α-helices and six β-strands that can undergo significant structural changes (Figure 1c & Movie 1). In all NP structures, the first fifty amino acids (α1, α2) involved in the multimerization mechanism [34] are folded over the core domain, suggesting a mechanism reminiscent of the one described in *Phenuiviridae* (see below). In the original structures of Qi and collaborator [32], NTP were observed to be trapped within the N-terminal domain. That result suggested a gating mechanism was allowing the access to a potential cavity. Instead, it misled the authors to propose that NP has potential cap-binding activity that could provide the host-derived primers to initiate transcription by the virus polymerase [32]. However, later studies showed that this putative NP cap-binding domain corresponded to the NP RNA binding site and the cap binding domain was identified in the C-terminal part of L protein [35,36,37]. The RNA binding cleft is indeed covered by two helices (α5, α6) and a loop (Figure 1c). A superposition of the structures alone or in complex with a ssRNA clearly presents the change of position of these latter secondary structure elements to create the cleft accommodating the RNA (Figure 1c and Figure S1a and Movie 1). In the RNA bound form, several positions of the helix (α6) are observed showing a large degree of liberty in its positioning (7 different positions are observed) [35].

The binding of the RNA and the opening of the cleft impact the secondary structure elements forcing the repositioning of the C-terminal domain to a hypothetical and not yet characterised position [35] (Figure S1b).

**Figure 2:**
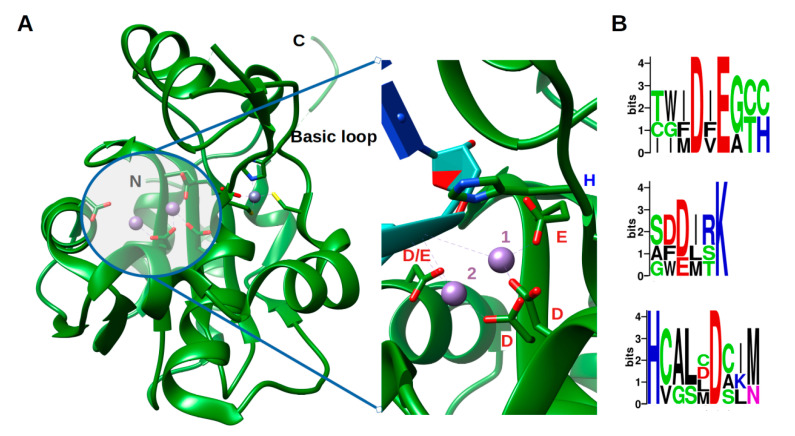
Structure of the exonuclease domain (ExoN) and conservation of the catalytic site. (**A**) Annotated structure of the ExoN domain represented in ribbon with ions Mn^2+^ in purple and Zn^2+^ in grey and zoom on the catalytic residues DEDDh shown in sticks with catalytic ions and 3′ end of double-stranded (ds)RNA substrate (cyan) (PDB). Metallic ion Mn^2+^ are marked as 1 and 2, 1 being the ion that is always observed and 2 the ion dynamically brought by the RNA. (**B**) Weblogo [51] of the DEDDh catalytic site through *Arenaviridae*.

Arenavirus NP, unlike other RNA negative stranded viruses (NSV) nucleoproteins, has acquired a 3′-5′ exonuclease domain specific to dsRNA in its C-terminal, exhibiting a type I interferon (IFN-I)-counteracting activity [38,39,40]. Moreover, this domain does not seem to be required for replication or transcription of the viral genome. This assertion, however, needs to be carefully evaluated as PICV, LASV and LCMV with a mutant NP lacking the 3′-5′ exonuclease activity had either not rescuable, impaired replication or a significant decrease in fitness during its replication [38,41,42]. Moreover, a recent in vitro study has shown a removing capability of mismatched nucleotide, which could be a first step in an editing process [43]. The ExoN belongs to the DEDDh family, that process their substrate through a two-metal ion catalytic mechanism [44], the two ions being coordinated by the residues of the motif. A sequence analysis of the ExoN domain of *Arenaviridae* shows the evolution of the catalytic motifs DEDDh to DEEDh (Figure 2 and Figure S2), an observation that added to the topology and the in vitro activity conservation with the ExoN domaine of *Coronaviridae* Nsp14, suggesting a common origin of the *Coronaviridae* nsp14 and *Arenaviridae* NP ExoNs [43,45]. This domain has a canonical fold of the DEDDh family of 3′- 5′ exoribonucleases, consisting of two β-sheets (with six mixed strands and two anti-parallel strands) and eight α-helices connected by a series of loops. These secondary structure elements are arranged to form the central β-sheet sandwiched by three α-helices on one side and seven α-helices on the opposite side and structured by a Zn binding site highly conserved in arenaviruses (Figure 2a). The reported structures often present one metallic ion in the catalytic site [46,47,48,49,50], the second ion, allowing the catalytic reaction, comes dynamically with the RNA substrate (Figure 2a zoom) [50]. Two flexible regions are clearly defined within the structure. The ‘basic loop’ sometimes structures itself as two anti-parallel strands (residues 514–526) above the active site and the C-terminal arm (residues 549–570). It is worth noting that in the full length structure, the latter region was folded over, between the NP-core and ExoN, while in ExoN domain structure, observation of crystal packing reveals that the C-terminal extends away from the domain core towards the back of the next ExoN core. Sequence alignment analysis shows a conserved hydrophobic patch at the C-terminal (Figure S3). This suggests that the C-terminal could also be involved in stabilizing the ExoN domain within the polymer assembly and packing (Figure S3). 

### 2.2. From Filament to Polymer Assembly 

**Figure 3:**
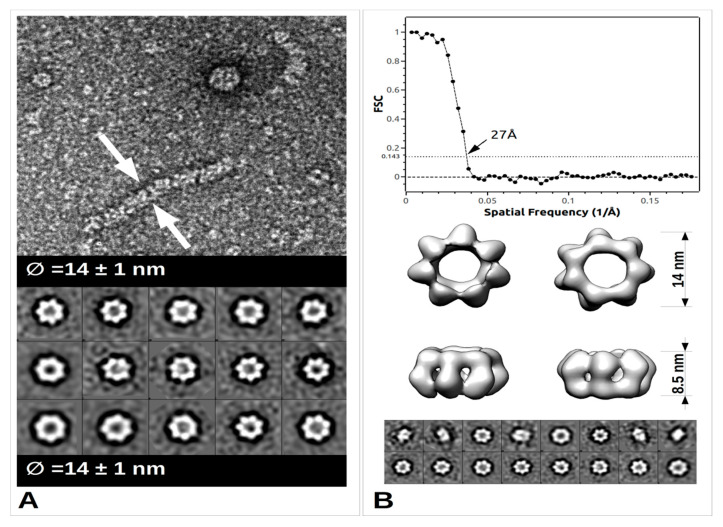
TEM images and data from freshly purified Mopeia virus (MOPV) NP protein. (**A**) Ribonucleoprotein complex (RNP) particle and several classses showing heptamer organisation (0.05 mg/mL). A 5 μL drop was applied to a freshly deposited and glow-discharged formvar-carbon-coated grid (Copper 300). The grid was stained with Nano-W^®^ (Nanoprobes) and transferred into a Tecnai 120 kV Electron Microscope. A total of 100 raw images were recorded with an EAGLE 2k × 2k CCD camera. Images were under-focused at 1–2 μm with a final resolution of 2.8 Å/pix. Boxing, classification, initial model calculation, as well as refinement for 3D reconstruction, was done with the EMAN2 pipeline [53]. Arrows indicate the sides of the measured object. (**B**) Top: Graph of the Fourier Shell Correlation (FSC) coefficient in function of the spatial frequencies in Å^−1^, arrow indicating the maximum resolution; Central: 3D reconstruction at 27Å resolution with below corresponding particle classes used (1224 particles).

The arenavirus RNP structural data are sparse, yet in the light of recent data, extremely informative on its general assembly [52]. The low-resolution EM structure of PICV RNP shows that it is mostly formed by a flexible structure composed of NP monomers assembled linearly, and forming a filament. This filament appears to fold progressively through a number of intermediate helical structures, that reveal an increasing number of NPs associated with each turn of the helix. They range from a fragile configuration of two to three NPs per turn to a more stable fibre-like structure in which the number of NPs could not be resolved [52]. Furthermore, additional packaging levels were observed with the presence of supercoiled structures [52] forming fibres with a diameter of 15 nm. From more recent EM studies, these trimeric assemblies were rediscovered and combined with high resolution crystallographic data [33]. Moreover, from bacterially- expressed and purified NP of Mopeia virus (MOPV), we have recently measured the full length MOPV N protein by negative stain Transmission Electron Microscopy (TEM). This offers some new results concerning the multimerization shown in Figure 3 (preliminary data and unpublished observation). These RNP particles were observed with a diameter 14 ± 1 nm as well as symmetric circular heptamers of the same diameter (Figure 3a), a result consistent with the original measurement of PICV RNP purified from the virus (Figure 1c of [52]). Unfortunately, near atomic cryo-microscopy data on these polymers are missing to place the packaging position of both domains in the RNP. However, using the observed particles, we have undertaken a low resolution 3D particle reconstruction using the EMAN2 pipeline procedure [53]. The result is shown in Figure 3b together with the corresponding Fourier Shell Correlation (FSC) coefficient in function of the spatial frequencies in Å^−1^ units and particle classes used in the refinement. This multimerization trend is reminiscent of the original observation [52] but differs from the trimeric NP complexes previously observed [33,35], and additional observations are needed in order to understand the multimerization mechanism of the NP. The various multimerization fashions reported may concern the RNP structure, or the NP in solution, or during the intracellular stage of NP accumulation, or interaction with cellular cofactors recruited by the NP before assembly into RNP polymers [35].

Let’s also note that, the above TEM observation of the RNP complex, is in line with observations concerning polymerisation in the case of *Bunyavirales* RNP [54,55,56].

## 3. A Phosphorylation Signal Controls NP Assembly and RTC Formation

Arenavirus RTC are structured by the NP [52,57,58]. While the first RTC comes from the virion, all the subsequent RTC have to assemble in the cytoplasm. As previously mentioned, NP assembly and RNA binding behaves differently depending on the context of infected cells, or if obtained from the protein production system. A recent study [59] suggests that RTC nucleation is regulated by the phosphorylation of a single conserved residue in the NP (T 206 in LCMV). A point mutation preventing phosphorylation suppresses the formation of RTC, while a mutant still allowing phosphorylation does not impact the RTC formation. Surprisingly, a mutant mimicking constitutive phosphorylation generated a more diffuse number of large RTC. The mechanism by which NP nucleates RTC is still unknown but these data suggest that a transient phosphorylation on a single amino acid of the NP allows the nucleation of the NP, thus initiating the RTC formation. Mechanistically, it means that the phosphorylation of the threonine just below the first amino terminal helix allows the polymerisation of NP to happen. As shown for various nucleoproteins, NP exists in at least two conformations (open/closed) implying a triggering signal to open the arm responsible for multimerization. Analysis of available Arenavirus NP sequences shows that at the equivalent position of LCMV T206, the threonine is strictly conserved in Mammarenavirus and predominantly replaced by a Glutamine, (followed by Serine, Alanine, or Methionine) in the other three genera (Figure S4).

This is not an uncommon mechanism in RNA negative-stranded viruses; for example, the NP phosphorylation of rabies virus (*Rhabdoviridae*) plays an important role in the regulation of viral transcription and replication, as phosphorylated NP are unable to encapsidate the rabies virus leader RNA. The regulation of NP polymerisation for RNA replication packaging is, therefore, controlled through transient NP phosphorylation [60,61]. A similar mechanism was observed for influenza A virus (*Orthomyxoviridae*), measles virus (*Paramyxoviridae*) and Marburg virus (*Filoviridae*) [62,63,64,65]. For all these viruses, the phosphorylation of the NP inhibited the transcription of viral RNA and prompted the polymerization of NP (in other words, switching to a replicative mode). However, this assumption for Arenaviruses prompts further structural and mechanistical investigation.

## 4. Arenavirus NP Assembly Compared to those of other *Bunyavirales* Nucleoproteins: The Brothers in Arms

Nucleoproteins in NSVs are the main viral protein of RNPs [28]. They have different modes and triggers for assembly, and apparently different packaging. Most of the characterised structures encapsidate the vRNA within, and few, on the contrary, act as a central support around which vRNA is wrapped [66]. The first type of packaging necessitates a local unwinding mechanism by the L itself and is sometimes assisted by a co-factor, while the second type of packaging only necessitates the RNA to transiently exit the RNA cleft to be processed by L. This packaging mechanism is, therefore, reflected in the mechanism of polymerization of the NP. The *Bunuyavirales* RNPs purified from virions are, more or less, all looking like twisted filaments without presenting the apparent helicity of tubular structures. The RNA segment is pseudo-circularised by interaction of its complementary base-pairing 5′ and 3′ ends, a structural motif recognized by the L protein (i.e the polymerase domain). It is presumed that this assembly occurs concurrently with genome replication, yet several have observed the spontaneous formation of RNP structures during NP expression, leading to the idea that the driving force of the assembly is, first and foremost, the proper physical environment (ionic strength, hydrophobicity, pH, concentration...) [57].

Comparison with other *Bunyavirales* nucleoprotein structures (with or without RNA) allows one to identify universal trends. Several structures of the full length nucleoproteins are now available from seven families of *Bunyavirales* including *Arenaviridae* (Lassa fever virus, LASV [32,33]), *Hantaviridae* (Hantaan virus, HTNV [67,68], Andes virus, ANDV [69], Sin Nombre virus, SNV [69]), *Nairoviridae* (Crimean-Congo haemorrhagic fever virus, CCHFV [70], Hazara virus, HAZV [71,72], Kupe virus, KV, Erve virus, EV [71]), *Peribunyaviridae* (LaCrosse Virus, LACV [73], Bunyamwera, BUNV [74,75], Schmallenberg virus, SHMV [75,76,77] and Leanyer virus LEAV [78]), *Phenuiviridae* (Rift Valley Fever Virus, RVFV [54,55,79], Toscana virus TOSV [54,56]), and *Tospoviridae* (Tomato spotted wilt virus TSWV [80]). 

**Figure 4:**
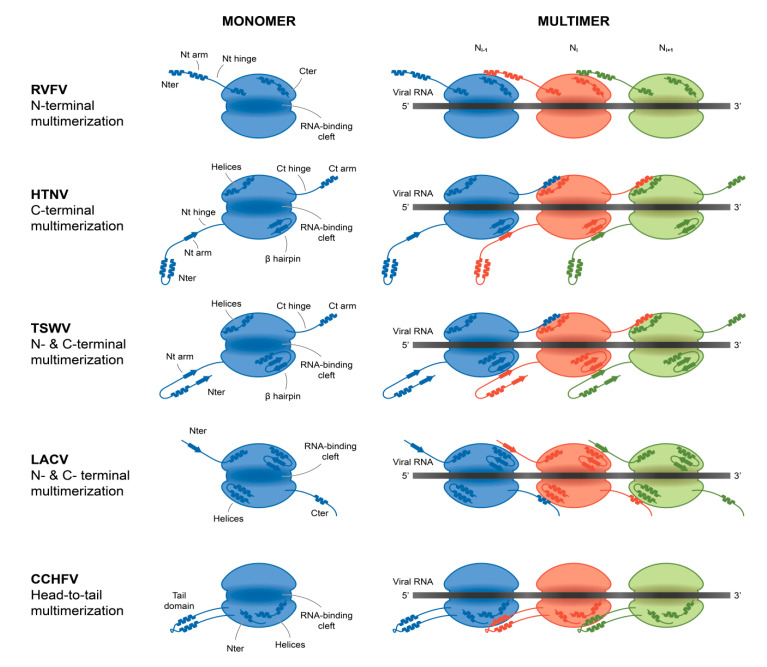
The schematics of slight increase in complexity of NP assembly. Major secondary structures involved in interprotomer interactions are represented as arrows for β-strand and cylinders for α-helices. The RNA binding cavity is represented as a central shaded part in the middle of the NP-core (blue macaron). Schematics representation from top to bottom of Rift Valley Fever Virus (RVFV)-NP, Hantaan virus (HTNV)-NP, Tomato spotted wilt virus (TSWV)-NP, LaCrosse virus (LACV)-NP, Crimean Congo Haemorrhagic Fever virus (CCHFV)-NP. Schematic representation of RNA binding and NP–NP interactions. RNA is shown as a black line. The main NP–NP interactions between adjacent subunits are indicated. For clarity, Ni interactions with Ni-2, Ni-3, Ni+2 and Ni+3 are absent from the schematic representation.

All the nucleoprotein structures present a globular core, which, in its middle, generally harbours an RNA binding cleft. In spite of limited sequence similarity, the RNA binding cleft can be identified by an enriched lysine, arginine (K/R) strip that guides the negatively charged phosphate backbone of the RNA and, in the case of *Peribunyaviridae*, with additional non-specific interactions with RNA riboses and bases. From the core domain protrudes either a single N-terminal (*Phenuiviridae*) or both N- and C-terminals (*Hantaviridae*, *Peribunyaviridae*, *Tospoviridae*), or else central (*Nairoviridae*) multimerization extensions, revealing a variety of oligomerization modes schematically summarized in Figure 4.

### 4.1. The Lateral Arm(s) Multimerization Domain

**Figure 5:**
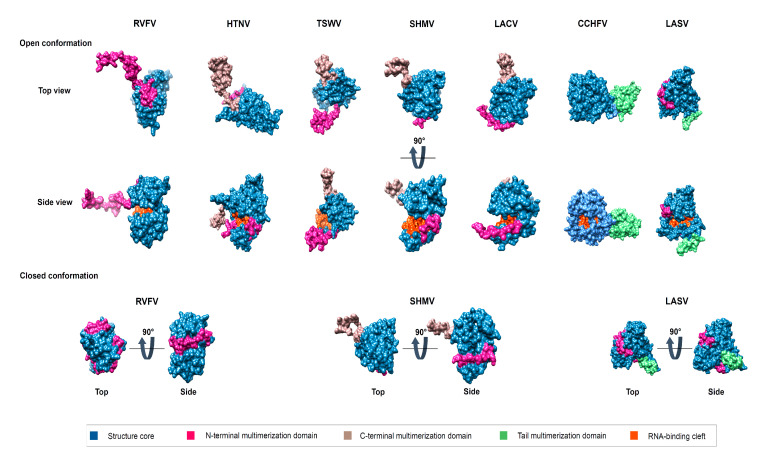
Structural comparison of known *Bunyavirales* NP. Surface representation of the different structures of representative *Bunyavirales* from left to right *Phenuiviridae* (RVFV, open conformation PDB: 3OUO /Closed conformation PDB: 3LYF), *Hantaviridae* (HTNV, PDB: 5FSG), *Tospoviridae* (TSWV, PDB: 5IP1), *Peribunyaviridae* (SHMV, open conformation PDB: 4DIX /Closed conformation PDB: 4DIU, LACV PDB: 4BHH), *Nairoviridae* (CCHFV, PDB: 4AQF) and, *Arenaviridae* (LASV core domain, open conformation PDB: 3T5Q /Closed conformation from PDB: 3MWP). Highlighted are the 3 important parts of the NP core in blue, multimerization arms N-terminal in pink, central in green, and the C-terminal in kaki.

*Phenuiviridae* nucleoprotein (RVFV, TOSV) (Figure 5) mediates its multimerization by the extended N-terminal arm folding around the surface of the core domain of the neighbouring protomer opposite to the RNA binding cleft. Several studies have shown that the arm is flexible, allowing complete closure of the RNA binding cleft to different conformations in the multimer, thus enabling the core domain to accommodate the physical constraint of the polymer [54,55,56,79]. In the polymer, the RNA binding cleft is inside, ensuring complete protection of the RNA (Figure 4).

*Hantaviridae, Peribunyaviridae* and *Tospoviridae* nucleoproteins present similar topological formations *per se*, a central core with a positively charged cleft to accommodate the vRNA from which extends two N- and C- terminal arms. Although the core is fairly structurally conserved between the families, the mode of assembly is specific (Figure 5).

*Peribunyaviridae* (LACV, SBV, LEAV) and *Tospoviridae* (TSWV) nucleoproteins mediate their mutltimerisation using their N- and C- terminal arms interacting with the surface of the core domain of the two neighbouring monomers in a head-to-tail pattern. The binding of the arms is opposite to the RNA binding cleft which, from one monomer to the next, forms a coherent continuous channel for accommodating the RNA. Each core is thus tightly bound and the vRNA is also inside the polymer (Figure 4).

In a recent cryo-electron microscopy study by Arragain et al. on *Hantaviridae* nucleoprotein (HTNV [68]) (Figure 5), the authors were able to reconstitute the 3D structure of the RNP, accessing the proper placement of the NP monomers [67]. The contact between monomers is ensured by exchange of their N- and C- terminal arms that make intimate contact with the core domain of neighbouring monomers. The N-terminal arm is a flexible elongated structure binding a β-hairpin that protrudes from the core of the previous neighbouring monomer, forming a 3-stranded β-sheet. The C-terminal arm binds a hydrophobic pocket of the following monomer. Both interactions are critical for multimerization [67,81,82]. Moreover, the two arms are joining in a clamp manner perpendicular to the RNA binding cleft. The surface in a shape of a triangle formed by the arms constitutes the interacting surface for the equivalent surface of the next monomer. In the polymer, the RNA binding cleft is inside the polymer, ensuring a complete protection of the RNA (Figure 4). It is worth noting that, contrary to other NP from *Phenuiviridae, Peribunyaviridae* and *Tospoviridae*, the RNA binding cleft is not continuously covered by the core but rather progresses in steps with flexible loops covering the interstitial gaps (Figure S5).

Different complex structures of NP RNA are proposed, in which the core is able to cover six to seven nucleotides (nts) for *Arenaviridae*, seven nts for *Phenuiviridae,* 11 nts for *Peribunyaviridae*, and six to eight nts for *Tospoviridae,* while HTNV structure would only cover three nts. These numbers are in fact estimates that need to be taken with caution, as previously discussed in [83]. Moreover, the reported crystallographic NP–RNA complexes *Phenuiviridae, Peribunyaviridae* and *Tospoviridae*, regardless of their mode of association, differ considerably, from trimer to higher oligomers. These intermediates are biologically irrelevant as they all lead to a circular encapsidation and not a twisted one, allowing the polymer formation with a continuous and long RNA. Yet all these structures present key elements such as the structural extension and its intrinsic flexibility to interpret the potential mechanism of assembly. Most likely, the observed differences are caused by the differences in RNA length and, to a lesser extent, sequence, different nucleoprotein preparation protocols, the oligomeric state of purification and stability, as well as crystallization conditions selecting one multimer as nucleus over others. 

### 4.2. The Central Multimerization Arm

In *Nairoviridae* (CCHFV) (Figure 5), NP possesses a racket-shaped structure [84]; however the final assembly with RNA path is still under investigation. Macroscopically, the polymers assemble as a double anti-parallel superhelix and the formation of the polymer is mediated by the interaction between the stalk domain of one monomer and the base of the head domain of the next monomer [70]. From the different structural observations, the two domains present a large degree of flexibility relative to each other, suggesting structural adaptation necessary for the polymer stability (for review [85]). Recently, two RNA-binding sites were recently identified, both in the head and stalk domains. At the positively charged cleft of the head domain, the protein is able to recognize the single strand RNA (ssRNA) [84], while a second RNA-binding site in the stalk domain can specifically recognize a panhandle structure, formed by the base-pairing of complementary nucleotides at the 5’ and 3’ termini of the vRNA genome [86].

**Figure 6:**
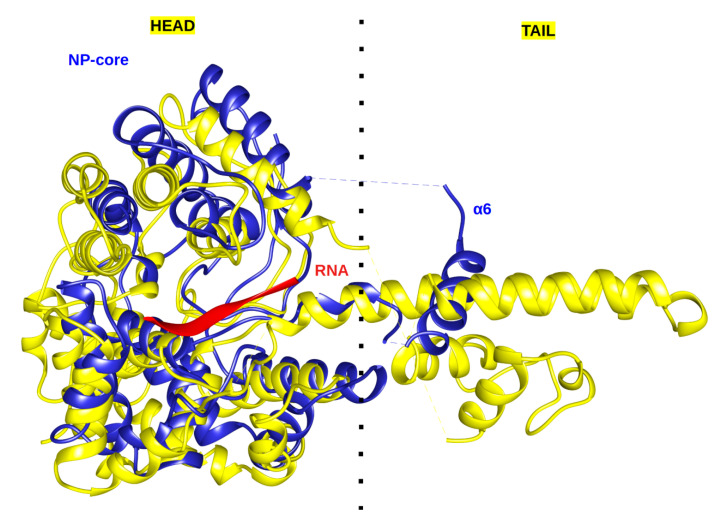
Structural comparison between the NP-core with RNA of LASV (blue, PDB 3T5Q) and the head domain of CCHF (yellow, PDB 3U3I) represented in ribbon. The two structures present a RMSD of 2.58 Å. The clear structural conservation between the two cores suggests that α6 is a valid structural candidate for multimerization.

Nairovirus NP is considered the most structurally distant NP from the *Phenuiviridae*, *Hantaviridae*, *Peribunyaviridae* and *Tospoviridae* previously mentioned. Interestingly, a structural homology search leads to the core of LASV as its closest homologue [84]. The structural comparison between Arenavirus and Nairovirus core domain structures, as shown in Figure 6, leads to the rational conclusion to assign the head domain as the NP-core with the RNA binding groove and the stalk domain as the multimerization domain, with its most likely one being the Arenavirus flexible helix α6. It was previously observed in crystal structure that α6 can be involved in crystal packing [35]. 

It is tempting to leap forward with the comparison of these structural similarities to a tentative model reconciling the structural data and proposing that arenvirus NP core domains have a side by side assembly, mediated by at least α6 and ensuring a continuous RNA binding cleft. However, the role and position of α1 and ExoN domains still need to be structurally determined, urging for new structural research on the topic.

## 5. Nucleoprotein Counteracting Innate Immune Response and Host Antiviral Defence

Immune response can be summarized into two categories (i) the adaptive and (ii) the host innate immune responses. The first one provides the host with a robust and long-term antiviral defence but needs days or weeks to reach its full potential. In this process, NP plays a critical role in adaptive immune responses. Indeed studies involving LF survivors and validated animal models of LASV infection showed that their CD4/CD8+ T-cells present responses against conserved NP’ epitopes [87,88]. This response is actively considered in LASV vaccine development by including both the GPC and NP in the formulation [89,90].

In contrast, the host innate response is quickly triggered at the cell level, providing protection and activating the subsequent adaptive immune response [91]. The control of the immunity by arenaviruses is a complex, finely tuned, and multifactorial process that goes beyond the scope of this review and involves several partners including the viral protein Z, as well as the type of cells in which the infection takes place (for review [92,93,94]), therefore we tried to focus on the direct implication of NP in the process. In spite of NP’s conserved domain organisation, atomic structure, and in vitro activity, significant phenotypic differences are observed from one arenavirus to the other [95], yet the IFN-I and the double-stranded RNA (dsRNA)-activated protein kinase (PKR) pathways are clearly identified as being targeted by the NP during arenavirus infection.

**Figure 7:**
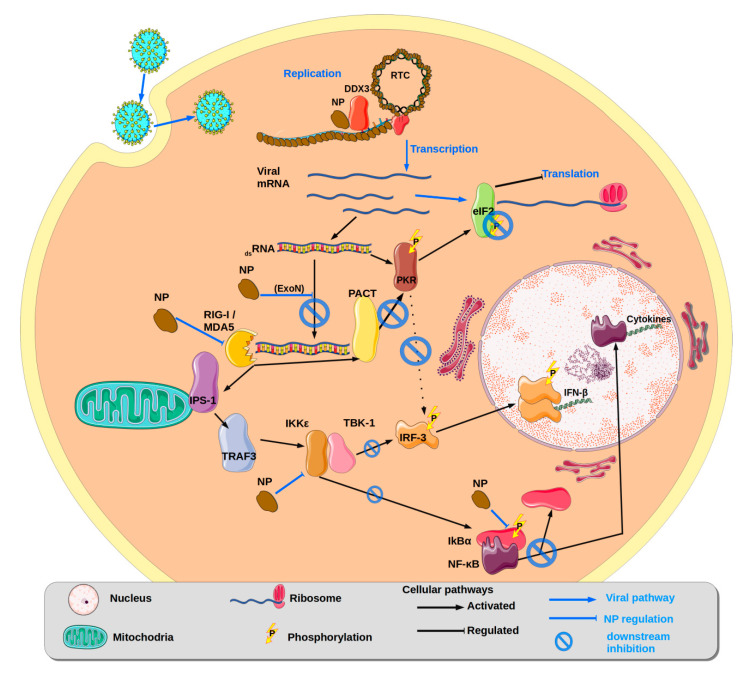
Pathways of innate immunity and its disruption by NP. Symbols are explained in the bottom caption.

IFNs play key roles in both the innate and adaptive immune response of the host against viral infections by establishing an antiviral state in infected and uninfected neighbouring cells; stimulating and regulating cells involved in innate and adaptive immunity such as NK cells, NKT cells, T cells, macrophages, and dendritic cells (DC) [96]. IFN-I can be induced by several classes of pattern recognition receptors (PRRs) (for review [97]). Among these RIG-I (retinoic acid-inducible gene-I), MDA5 (melanoma differentiation-associated gene 5) are prime targets of arenaviruses NP activity. RIG-I detects 5′-triphosphate single-stranded RNA (ssRNA) and short (<2 kb) double-stranded RNAs (dsRNAs) in most cell types, whereas MDA5 is responsible for the recognition of virus-derived, long (> 2 kb) dsRNA [97,98,99]. Upon binding to viral RNA, activated RIG-I and MDA5 transduce a signal to their downstream partners (for review [100]) thus activating the IKKε/TBK-1 (serine/threonine kinases IκB kinase ε/TANK-binding kinase-1) complex and the IKKα/β complexes [101,102,103]. In turn, these complexes phosphorylate IRF-3 (IFN regulatory factor-3) and NF-κB, which will undergo nuclear translocation to initiate the expression of IFN-β, IFN-α, and cytokines [104,105,106]. PKR is a well-characterised antiviral protein [107] that inhibits cap-dependent protein translation initiation via phosphorylation of the ribosome eukaryotic Initiation Factor 2 (eIF2α) once dsRNA is detected [108]. PKR can also be activated by the protein activator of the IFN-induced protein kinase (PACT) [109,110], and subsequently mediates the activation of the transcription factor NF-κB, by phosphorylating its inhibitory subunit [111] (Figure 7). 

Early studies on LCMV have demonstrated that the NP blocks the nuclear translocation and transcriptional activity of IFN regulatory factor 3 (IRF-3), which results in the robust inhibition of IFN-I production [40]. This IFN-counteracting activity is the result of upstream regulation of several cooperative processes, i.e., RNA degradation and protein sequestration (Figure 7) [112]: (i) The conserved ExoN domain degrades viral dsRNAs produced during viral replication, preventing their accumulation in the cytoplasm and the risk of recognition by non-self RNA sensors such as PKR, PACT, RIG-I and MDA-5 [113,114,115]. Consequently, RIG-I and MDA5 are not activating the cascade that allows the phosphorylation of IKKε that will trigger the activation of IRF3. Neither PACT nor PKR are activated, leading to the persistence of an active eIF2, allowing the translation to continue and the impairment of the activation of NF-kB [116]. In JUNV infection, this blockade of PKR function is highly specific and complete. On the contrary, LCMV NP is unable to similarly inhibit the eIF2α phosphorylation beyond 36 h post infection of LCMV [117]. The mechanism underlying dsRNA accumulation in JUNV and MACV infections is still unclear, as dsRNA is readily accumulated during JUNV and MACV infections or in JUNV minigenome replication studies. The ExoN activity of PICHV [41] and MACV have been determined in vitro and behave similarly to other OW ExoN [43]. The crystal structure study of the JUNV ExoN shows that the domain is conserved and similar to the ExoN of LCMV LASV, MOPV, TCRV [47,48,49,50]. However, the authors have failed to demonstrate the ExoN activity in vitro [46]. These data do not suggest an inactive dsRNA clearance mechanism but, rather, a rescue mechanism of the inhibited pathway in these viruses. A possible variation in ExoN activity or its regulation among NW arenavirus NPs also needs to be considered. Further studies are needed in this particular field. (ii) Additionally to the common mechanism of dsRNA clearance by the ExoN during Arenavirus infection, NP engages several proteins of the host, aiming at the disruption of several antiviral pathways. Moreover, the targeted cellular proteins seem to be the result of specific adaptations of each virus to their host as LCMV, JUNV, LASV or TCRV look to have exclusive targets [117,118,119]. LCMV NP was shown to specifically associate with the kinase domain (KD) of IKKε. The NP–IKKε interaction was highly conserved among arenaviruses from different clades and they block its autocatalytic activity and its ability to phosphorylate IRF3 [120].

Recently, NP of LCMV, LASV and JUNV were shown to interact with DDX3 [118]. DDX3 is a DEAD-box ATP-dependent-RNA-helicase [121] involved in different roles in RNA metabolism including transcription, translation, nuclear export, and assembly of stress granules. DDX3 is also a component of the innate immune response against viral infections playing roles both upstream and downstream of (IKKε)/TANK-binding kinase 1, leading to IFN-β production [122,123]. Interestingly, other RNA viruses, including HCV, HIV-1, JEV, WNV and HBV [121,124], were reported to hijack DDX3 to accomplish various steps of their replication cycles. In arenaviruses at early stage of infection, DDX3 would be recruited to assist in viral RNA replication, as supported by the reduced arenavirus minigenome replication in DDX3 knockout cells [118]. In late infection, however, DDX3 is proposed to act as an IFN suppressor. Moreover, in this pathway, different efficiencies are observed depending on the virus; as with LCMV, it does suppress IFN production-facilitating infection, while it fails to do so in JUNV.

In spite of the diverse panel of strategies deployed by arenaviruses, it seems that the IFN antagonistic activities of NP are not sufficient to abolish the host innate immune response, as, in the context of infection by OW and NW, arenaviruses such as IFN-I and cytokine are still induced [100,125,126,127,128,129]. 

The effect of the ExoN also impacts the immune response at a cellular level. The infection causes immunosuppression, due to the absence of activation of antigen-presenting cells (dendritic cells (DC) and macrophages), low type I interferon (IFN) production, and deficient NK cell activation. DC and macrophages are central players in the innate and adaptative host immune response that the virus needs to delay, insuring its survival. Although during arenavirus infection both types of cells are infected early, it is in DC that NP IFN antagonist activity seems prevalent [130,131]. The deficiency in NK cells response is a consequence of the ExoN activity, as shown in LASV infection defective of the ExoN activity. In this defective ExoN virus, the IFN-I is strongly induced in both DC and macrophages and triggers efficient human NK cell responses [132]. The effect of ExoN also reaches NK cells, explaining the weak NK cell activation observed with the wild-type virus at a cellular level. 

A final point to address is the complexity of arenaviral immuno-suppression effect, regarding pathogenesis and persistence. Potent suppression of innate and adaptive immune responses is the hallmark of severe LASV infections in humans. As mentioned previously, arenaviruses induce distinct IFN responses in human cells [129]. LASV productively infects human macrophages and dendritic cells but fails to activate these cells or induce significant amounts of IFN/cytokine expression. In addition, LASV infection poorly induces T cell proliferation [128]. In comparison, MOPV induces strong IFN/cytokine responses and grows poorly in human macrophages [130]. Therefore, the innate immune responses, including the IFN response, can be interpreted as critical to the control of nonpathogenic arenavirus infection. In contrast, the highly pathogenic LASV blocks host innate and adaptive immune responses and causes severe and often fatal diseases. It is also worth noting that in the case of LASV infection in nonhuman primate model, an early peak of type I IFN production correlated with animal survival, whereas fatal infection was characterized by a lack of early type I IFN production [133]. 

NP of TCRV, the only mammalian arenavirus isolated from non-rodent species, lacks anti-IFN activity [39] in spite of a conserved ExoN. This fact stresses the limited understanding of the role of the exoN activity, suggesting it has a broader involvement in the viral life cycle than expected. At this point, we can only propose that the ability of the viral NP to interfere with induction of the IFN-I systems may be a necessary but not sufficient factor in arenavirus virulence and probably involved in the persistent form of infection in natural hosts, which is another arenaviral common feature.

## 6. Conclusions

In spite of recent structural and functional advances, we still do not have a complete understanding of the final structure of the NP polymer assembly nor the trigger allowing the conformational changes that a single monomer undergoes to become a polymer upon binding RNA. We still hold that fragmented views and further structural investigation at multiple scales will be needed to be able to propose a functional packaging mechanism allowing the assembly of a functional RTC. Nevertheless, the accumulated structural data on *Bunyavirales* nucleoproteins allow the delineation of common structural patterns involved in multimerization. The implication of flexible protruding helices, terminal or central, seems to be the common mechanism to the nairo- and arenaviruses. Similarly, we also need a better understanding of the structural consequences of the NP phosphorylation and its regulation, which, if confirmed, could be key to stabilized RNP complexes. The functional implication of NP and its ExoN, interfering at so many levels in the immune response, makes the NP a valuable target for both vaccine and inhibitor design, specific to the nuclease activity and/or to the protein–protein interactions. This review lets us glimpse the dynamic nature of the NP system and we can hope for a better understanding of these changes with the use of time-resolved crystallography.

## Supplementary Materials

The following are available online at https://www.mdpi.com/1999-4915/12/7/772/s1. Figure S1: Gating mechanism of the RNA binding cleft. Focus on the RNA binding cleft, by comparing the RNA binding domain of the 3WMP structure with the corresponding domain of the structure 3T5Q. Figure S2: Observed interaction of the C-terminal ExoN (PDB 5LS4) and its next of keen suggesting hydrophobic interaction contributing in the multimerisation process. Figure S3: Best fit of the NP core leads to a continuous RNA binding cleft, position of RNA. Figure S4: NP-core structure with the phosphorylated Threonine highlighted in yellow and showing its critical position under helix α1. Figure S5: Stepping assembly of NP HTNV. A) Reconstruction of the HTNV filament from EM data and model 6I2N. Movie 1: Gating mechanism of the RNA binding cleft of LASV NP-core. All structural figures and movie were done using UCSF chimera [126].
