# Supplementary material for: Brothers in Arms: Structure, Assembly and Function of Arenaviridae Nucleoprotein
Source: Viruses. 2020 Jul 17;12(7):772. doi: 10.3390/v12070772 (PMC7411964; doi:10.3390/v12070772)
Supplement: Supplementary file 1 [file viruses-12-00772-s001.zip › viruses-852382-supplementary/viruses-852382-supplementary.docx]

Supplementary materials


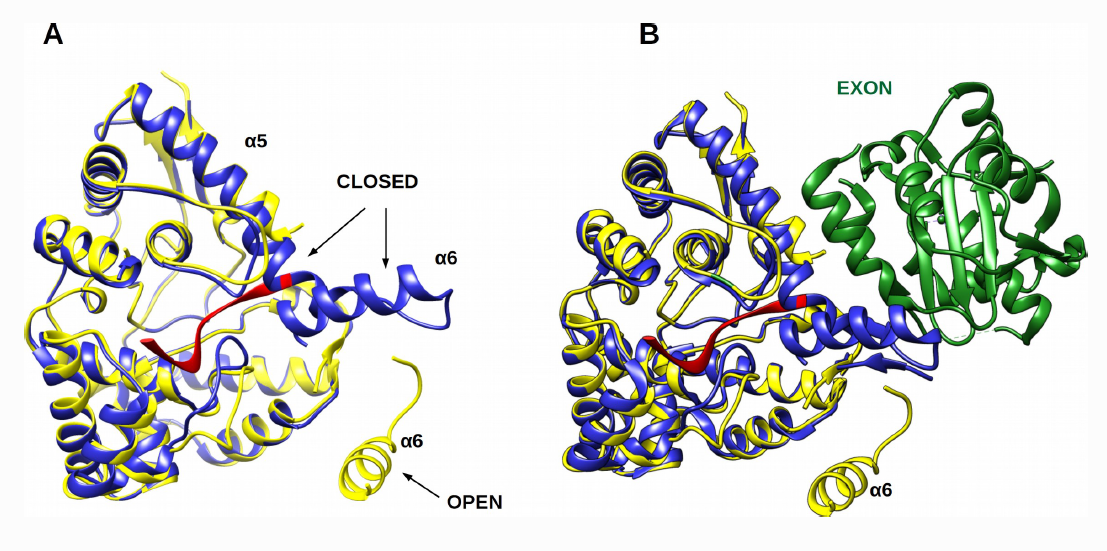


Figure S1: Gating mechanism of the RNA binding cleft. Focus on the RNA binding cleft, by comparing the RNA binding domain of the 3WMP structure with the corresponding domain of the structure 3T5Q. (a) A superposition of the two structures. The 3WMP structure is shown in green while the 3T5Q in yellow. In the later an RNA molecule of 6 nucleotides (red) is clearly positioned on the RNA binding cavity. Comparison of these two structures shows that in the absence of RNA (3WMP) the cavity is closed by the α5 and α6 helix shown in blue as well as by the loop residues 237 – 241 shown also in blue and noted as “L”. The α5 and α6 helix as well as the “L” loop are displaced in the case of the 3T5Q structure permitting the adsorption of the viral RNA. (b) Structural overlap of helix α6 and ExoN domain. The superimposition shows the position of the ExoN domain relative to the RNA binding domain of the N as measured in the full length NP structure 3WMP interrupting the continuity of the RNA cavity and structural conflict with helix α6. Thus it’s relative position has to be different within the RNP complex.


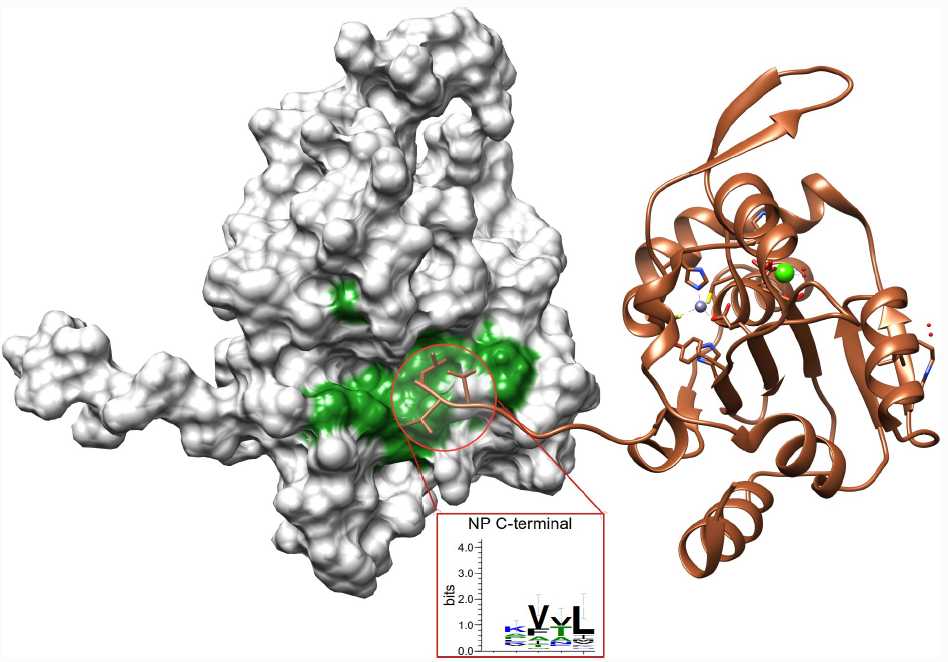


Figure S2: Observed interaction of the C-terminal ExoN (PDB 5LS4) and its next of keen suggesting hydrophobic interaction contributing in the multimerisation process. In box, the Weblogo analysis of the C-terminal show a conserved hydrophobic patch.


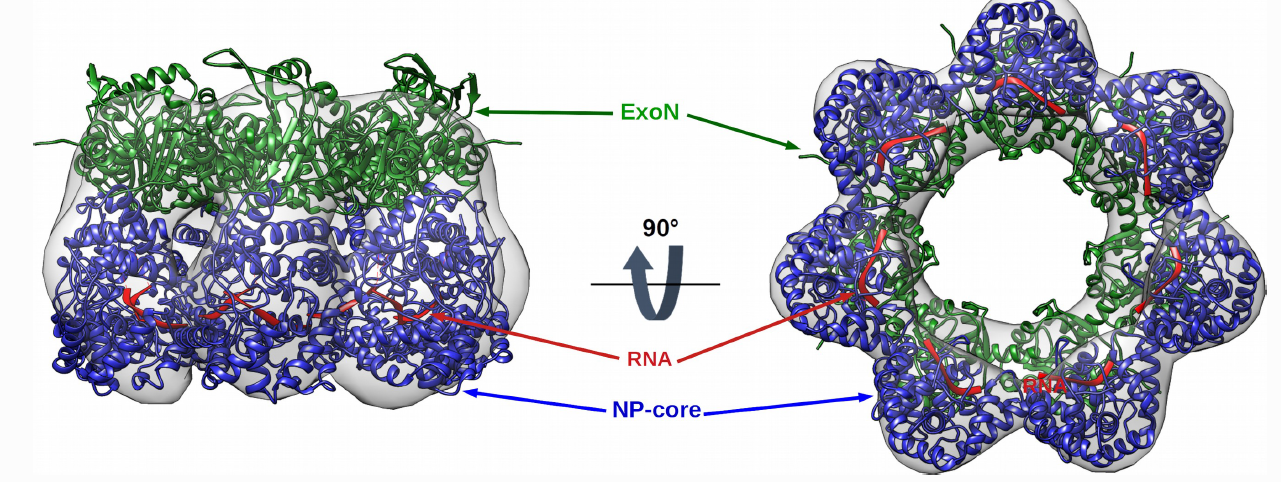


Figure S3: Best fit of the NP core leads to a continuous RNA binding cleft, position of RNA (red). 3T5Q model of NP-core structure (blue) and ExoN (green PDB 3Q7B) fitted into the measured 3D TEM electron density. Structure and surface rendering were done using [128]


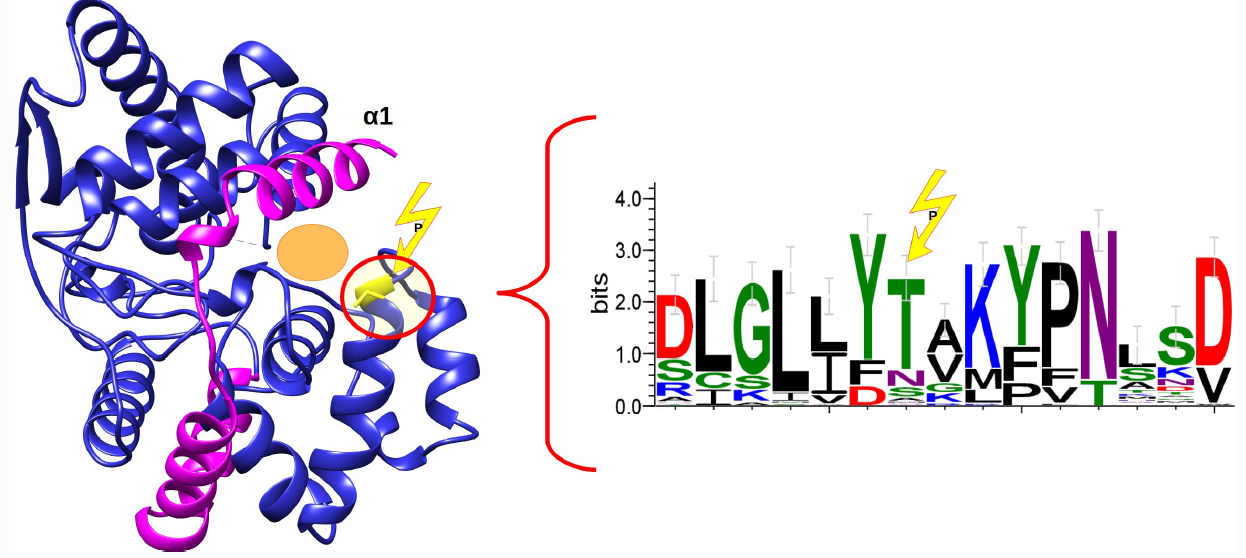


Figure S4: NP-core structure with the phosphorylated Threonine highlighted in yellow and showing its critical position under helix α1. Weblogo representing the position being phosphorylated in Arenaviridae regulating the RNP formation. Threonine is conserved in Mammarenavirus, while variation are observed in other genus suggesting different regulation due to host adaptation.


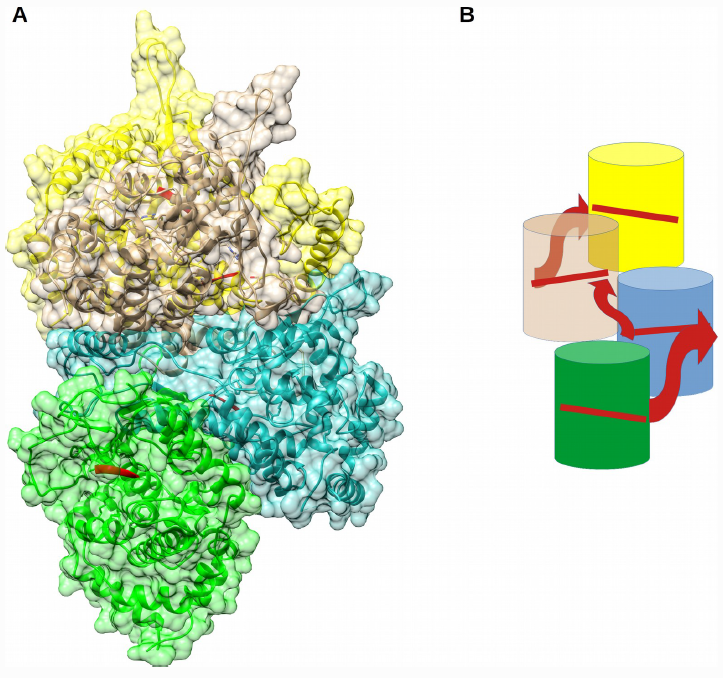


Figure S5: Stepping assembly of NP HTNV. A) Reconstruction of the HTNV filament from EM data and model 6I2N. RNA (red ribbon) located in binding cleft. 4 NP’ are represented in surface (green, blue, kaki, yellow). B) Schematic representation of A , resolved RNA is represented by a red stripe, and red arrow symbolised the putative path to recreate a continuous filament. The RNA cleft is not continuous but rather progress in steps.

Movie1: Gating mechanism of the RNA binding cleft of LASV NP-core. All structural figures and movie were done using UCSF chimera [126].
